# Protocol for recombinant expression in E. coli and purification of iBoost vaccine proteins using immobilized metal affinity chromatography

**DOI:** 10.1016/j.xpro.2026.104572

**Published:** 2026-05-19

**Authors:** Elisabeth J.M. Huijbers, Judy R. van Beijnum, Joost Koetsier, Gabriela E. Wachholz, Peter C.J. Kemper, Lauren Myburgh, Steijn Meister, Karlijn van Loon, Mattie A. Cassee, Arjan W. Griffioen

**Affiliations:** 1 Angiogenesis Laboratory, Department of Medical Oncology, Amsterdam UMC, Cancer Center Amsterdam, 1081 HV Amsterdam, the Netherlands; 2 CimCure BV, 1066 CX Amsterdam, the Netherlands

**Keywords:** Cell Biology, Cancer, Immunology, Molecular Biology, Protein Biochemistry, Protein expression and purification, Biotechnology and bioengineering

## Abstract

The iBoost vaccine technology enables the induction of a humoral immune response against self- and low-immunogenic antigens through fusion of the antigen to an immunogenic bacterial carrier. Here, we present a protocol for recombinant expression in *E. coli* and purification of iBoost vaccine proteins using immobilized metal affinity chromatography (IMAC). In addition, we describe procedures to evaluate antigen-specific immune responses in mice. This protocol can be readily adapted for the generation and testing of vaccines against a broad range of antigens.

For complete details on the use and execution of this protocol, please refer to Huijbers et al., Van Beijnum et al., and Van Loon et al.^1^^,^^2^^,^^3^^,^^4^

## Highlights

• Steps to clone, produce, and purify iBoost conjugate vaccine proteins in *E.coli*
• Protocol for vaccination and blood sampling of mice
• Evaluation of antigen-specific antibody responses in mice by ELISA

## Abstract (editor highlights)

Publisher’s note: Undertaking any experimental protocol requires adherence to local institutional guidelines for laboratory safety and ethics.

## Graphical abstract

**Figure:**
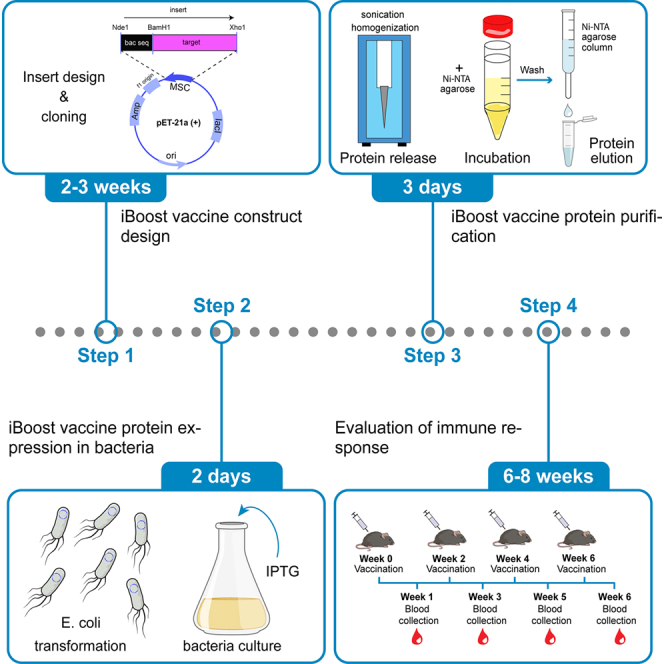

## Before you begin

This protocol describes a method for inducing robust antibody responses against self-antigens or poorly immunogenic foreign molecules, a strategy with therapeutic potential against disease, e.g., cancer, and prophylactic utility against cancer or infectious diseases. The approach is based on a next-generation conjugate vaccine platform known as immune Boost (iBoost) technology, which employs engineered *E. coli*-derived sequences, termed iBoost carriers. The latter provide T cell help while minimizing immunodominance and pre-existing immunity issues associated with traditional carriers, such as keyhole limpet hemocyanin or tetanus toxoid.

The workflow begins with the design of a vaccine construct, in which a self-antigen or low-immunogenic target is fused to an iBoost carrier such as truncated thioredoxin or a chimeric designer peptide.^1^ Codon-optimized constructs are cloned into bacterial expression vectors (e.g., pET21a), expressed in *E. coli*, and purified using Ni-NTA chromatography under denaturing or native conditions. Protein quality is assessed by SDS-PAGE, western blotting, and/or mass spectrometry. The purified conjugate protein is then formulated with a potent adjuvant, e.g., Freund’s Adjuvant or Montanide/CpG, and administered subcutaneously in mice at 10–14-days intervals (typically 3–4 doses of 100 μg each). The entire process, from design to *in vivo* validation, can be completed within 3.5 months, with immune responses detectable within 30 days after initial vaccination.

The procedures described here involve the use of genetically modified organisms (GMOs). A permit may be required for working with GMOs, and all relevant local laws and regulations must be followed. The microorganisms used in this protocol require Biosafety Level 1 (BSL1) facilities. Additionally, personnel should receive appropriate training, and aseptic techniques should be employed where applicable.

Mouse experiments should always be performed considering animal welfare according to national rules and regulations. Prior to execution of the mouse experiments an animal license from the national and local animal welfare bodies should be obtained. Additionally, personnel must be trained.

### Innovation

This method enables the targeted breaking of immune tolerance by genetically coupling low-immunogenic- or self-antigens to a highly immunogenic bacterial carrier, thereby providing a versatile and efficient platform for the development of therapeutic vaccines, as well as prophylactic vaccines, against a broad range of antigenic targets.

### Institutional permissions

All *E. coli* related work was conducted in BSL1 at Amsterdam UMC or CimCure BV with national permission for this research. Users must obtain appropriate training and approvals before working with *E. coli*.

All mouse experiments were performed in accordance with Dutch guidelines and law on animal experimentation and were approved by the Centrale Commissie Dierproeven (CCD). Work protocols were approved by the VU-VUmc animal welfare body.

## Key resources table

**Table:** 

REAGENT or RESOURCE	SOURCE	IDENTIFIER
**Antibodies**		

Polyclonal Goat Anti-Mouse Igs/Biotinylated Dilution 1:2000	Dako	Cat#: E0433
Goat Anti-Mouse IgG1, Human ads-BIOT Dilution 1:2500	Southern Biotech	Cat#: 1070-08
Goat Anti-Mouse IgG2a, Human ads-BIOT Dilution 1:2500	Southern Biotech	Cat#: 1080-08
Goat Anti-Mouse IgG2b, Human ads-BIOT	Southern Biotech	Cat#: 1090-08
Goat Anti-Mouse IgG2c, Human ads-BIOT Dilution 1:2500	Southern Biotech	Cat#: 1079-05
Goat Anti-Mouse IgG3, Human ads-BIOT Dilution 1:2500	Southern Biotech	Cat#: 1100-08
Goat Anti-Mouse IgM, Human ads-BIOT Dilution 1:2500	Southern Biotech	Cat#: 1020-08
Streptavidin/HRP dilution 1:2000	Dako	Cat#: P0397

**Bacterial and virus strains**		

*Escherichia coli* BL21 (DE3)	Sigma-Aldrich	Cat# 69450
*Escherichia coli* TOP10	Invitrogen	Cat# C404010
*Escherichia coli* Rosetta gami (DE3) (Novagen)	Sigma-Aldrich	Cat# 70954

**Chemicals, peptides, and recombinant proteins**		

Ampicillin sodium salt	Merck	Cat# A9518-5G
Isopropyl-b-D-thiogalactopyranoside (IPTG)	Serva	Cat# 26600.04
Ethylenediaminetetraacetic acid disodium salt dehydrate (EDTA)	Sigma-Aldrich	Cat# E5134
N-Lauroylsarcosine sodium salt	Sigma-Aldrich	Cat# L5125
0.1 M Phenylmethanesulfonyl fluoride solution (PMSF)	Sigma-Aldrich	
Urea, 99.5%, for analysis	Acros Organics	Cat# 10665572
NaCl	VWR	Cat# 27788.366
NaH_2_PO_4_	Merck	Cat# 1.06346.1000
Phosphate Buffer Saline (PBS), pH 7.4, 500 mL	Thermofisher Scientific	Cat# 10010023
Ni-NTA Agarose	Qiagen	Cat# 30210
100GR Imidazole BAKER ANALYZED Reagent	JT Baker	Cat# 1747.0100
Trizma base	Merck	Cat#T1503
Hydrochloric acid fuming 37%	Merck	Cat#1.00317.0510
Glycerol	VWR	Cat# 24386298
Novex™ Tris-Glycine Mini Protein Gels, 4–20%, 1.0 mm, WedgeWell™ format	Thermofisher Scientific	Cat# XP04200BOX
Novex™ Tris-Glycine SDS running buffer (10×)	Thermofisher Scientific	Cat#LC2675-4
Novex™ Tris-Glycine SDS sample buffer (2×)	Thermofisher Scientific	Cat#LC2676
NuPAGE™ Sample Reducing Agent (10×)	Thermofisher Scientific	Cat# NP0004
Spectra™ Multicolor broad range protein ladder	Thermofisher Scientific	Cat#26634
TWEEN 20	Sigma-Aldrich	Cat# P7949
Triton X-100	Merck	Cat# X100-500ML
Triton X-114	Merck	Cat#X114
KH_2_PO_4_ potassium phosphate monobasic	Sigma-Aldrich	Cat# P0662-500G
K_2_HPO_4_ (di-potassium hydrogen phosphate)	Merck	Cat# 1.05104.1000
Tryptone vegetable	Merck	Cat# 16922-500G-F
Yeast extract	Merck	Cat# Y1625-1000G
Coomassie brilliant Blue G CBB-G250	Serva	Cat# 17524.02
Phosphoric acid 85%	ThermoFisher	Cat# 29570010
Ammonium sulfate	VWR	Cat#21,333,365
Methanol	VWR	Cat#2090.368
Freund’s Adjuvant Complete	Sigma Aldrich	Cat# F5881-6x 10mL
Freund’s Adjuvant Incomplete	Sigma Aldrich	Cat# F5506-10x 10 mL
Blotto, non-fat dry milk	Santa Cruz	Cat# sc-2324
Horse serum (H1138), heat inactivated	Sigma-Aldrich	Cat# H1138
3,30,5,50-Tetramethylbenzidine (TMB) Liquid Substrate System for ELISA	Sigma-Aldrich	Cat# T0440
Silde-A-Lyzer G3 dialysis cassette	ThermoFisher Scientific	Different MWCOs available
Montanide ISA 720	Seppic/Fisher Scientific	Cat# NC0685296

**Critical commercial assays**		

Plasmid isolation Midi kit	Qiagen	Cat#: 12143
Micro BCA Protein Assay Kit	Thermo Scientific	Cat#: 23235

**Experimental models: Organisms/strains**		

Mus musculus, C57BL/6OlaHsd wild type, male/female, 6–8 weeks old	Inotiv	N/A
Mus musculus, BALB/cOlaHsd wild type, male/female, 6–8 weeks old	Inotiv	N/A
Mus musculus, C3H/HeNCrL wild type, male/female, 6–8 weeks old	Charles River	N/A

**Oligonucleotides**		

CpG1826 T∗C∗C∗A∗T∗G∗A∗C∗G∗T∗T∗C∗C∗T∗G∗A∗C∗G∗T∗T	Eurogentec	Van Loon et al. Cancers 2022

**Recombinant DNA**		

pET21a(+) plasmid DNA – Novagen	Merck	Cat# 69740

**Other**		

KA INC 125 FS Digital (SP25) incubator	IKA	Cat#L030117230
OD600 DiluPhotometer	Geneflow	Cat#SA-0600
Soniprep 150 Ultrasonic Disintegrator MSE	MSE	N/A
LM10 Microfluidizer Processor – high shear laboratory homogenizer	Mircofluidics	Cat#MFLM10
Glass Microfiber Filters/Grade 13400	Sartorius	Cat# 13400-100------K
B Braun Injekt Solo Cone Syringes 2mL	Fisher Scientific	Cat# 12722637
B Braun Omnifix 100 Solo 1mL Insulin Syringe	Fisher Scientific	Cat# 15154541
Magnetic stirrer, ESP, Velp Scientific	VWR	Cat# 442-118
Tecan Infinite 200 Pro	Tecan	N/A
Vortex Genie 2 mixer	Merck	Cat#Z25423
Head set for Vortex-Genie	Merck	Cat#Z258431
Mline®Mechanical Pipette, 12 channel, 10-100 μL	Satorius	Cat#725230
Mline®Mechanical Pipette, 12 channel, 30-300 μL	Satorius	Cat#725240
Digital Heatblock	VWR	Cat#460-0349
Mini Gel Tank	Thermofisher Scientific	Cat# A25977
Parafilm M	Sigma Aldrich	Cat# P7793
BD Plastipak syringe 1mL, 3-Piece, luer-lock	Becton Dickinson	Cat# 309628
BD Microlance needles, 23G ×1″, blue, 0.6x25 mm	Becton Dickinson	Cat# 300800
BD Microlance needles, 25G ×5/8″, orange, 0.5x 16 mm	Becton Dickinson	Cat# 300600
Brand ® micro haematocrit capillary	Merck	Cat#BR749311-1000EA
Blood collection microtubes	SAI Infusion Technologies	Cat#MV-S-300
Microvette® CB 300 Serum CAT, 300 μl	Sarstedt	Cat#16.440
Clear Flat-Bottom Immuno Nonsterile 96-Well Plates	Thermo Fisher Scientific	Cat# 442404
Easy seal	Greiner Sigma-Aldrich	A5596-100EA Cat#676001
Rocker 2D digital	IKA	Cat#0004003000

## Materials and equipment

**Table:** LB culture medium (1 L)

Reagent	Final concentration	Amount
Tryptone	10 g/L	10 g
Yeast extract	5 g/L	5 g
NaCl	10 g/L	10 g
ddH_2_O	N/A	∼975 mL
**Total**	**N/A**	**1 L**

Store at 2–8°C for up to 6 months. After opening store at 15–25°C for up to 1 month.

### Preparation of LB culture medium (1 L)

**Timing: 3 h**

• Weigh 10 g tryptone, 5 g yeast extract and 10 g NaCl and add to a 1 L glass bottle.
• Fill the bottle up to 1 L with dd H_2_O.
• Sterilize by autoclaving for 20 min at 15 psi (1.05 kg/cm^2^) on liquid cycle.

**Table:** TB culture medium (1L) – tryptone yest solution (900 mL)

Reagent	Final concentration in 1 L TB	Amount
Tryptone	12 g/L	12 g
Yeast extract	24 g/L	24 g
Glycerol	0.4% v/v	4 mL
ddH_2_O	N/A	900 mL
**Total**	**N/A**	**900 mL**

Store at 2-8°C for up to 2 months.

**Table:** TB culture medium (1L) – 0.17 M KH_2_PO_4_, 0.72 M K_2_HPO_4_ solution (100 mL)

Reagent	Final concentration in 1 L TB	Amount per 100 mL
KH_2_PO4	2.31 g/L	2.31 g
K_2_HPO_4_	12.54 g/L	12.54 g
ddH_2_O	N/A	∼100 mL
**Total**	**N/A**	**100 mL**

Store at 2-8°C for up to 2 months.

### Preparation of terrific broth culture medium (1 L)

**Timing: 3 h**

#### Preparation of tryptone yeast solution (900 mL)

• Weigh 12 g tryptone and 24 g yeast extract and add to a 1 L glass bottle.
• Add in 900 mL ddH_2_O and 4 mL glycerol.
• Shake or mix on a magnetic stirrer until the solutes have dissolved.
• Sterilize by autoclaving for 20 min at 15 psi (1.05 kg/cm^2^) on liquid cycle.
• Allow the solution to cool to 60°C or less, and then add 100 mL of a 0.17 M KH_2_PO_4_, 0.72 M K_2_HPO_4_.

#### Preparation of a 0.17 M KH_2_PO_4_, 0.72 M K_2_HPO_4_ solution (100 mL)

• Weigh 2.31 g of KH_2_PO_4_ and 12.54 g of K_2_HPO_4_ and add to a 100 mL glass bottle.
• Add 90 mL of ddH_2_O.
• After the salts have dissolved, adjust the volume of the solution to 100 mL with ddH_2_O.
• Sterilize by autoclaving for 20 min at 15 psi (1.05 kg/cm^2^) on liquid cycle.

**Table:** Colloidal Coomassie stain (1L)

Reagent	Final concentration	Amount
Serva Blue G solution	5% w/v (50 mg/mL)	20 mL
Phosphoric acid 85% v/v	N/A	11.7 mL
ddH_2_O	N/A	980 mL
**Total**	**N/A**	**1 L**

Store at 15-25°C for up to 1 year.

### Preparation of colloidal Coomassie stain (1 L)

**Timing: 1 h**

• Prepare a 5% (wt/vol) Serva Blue G solution.
  ○ Weigh 2.5 g CBB G-250 and add to a 50 mL Falcon tube.
  ○ Add 50 mL of ddH_2_O.
• Add 11.7 mL 85% phosphoric acid to 980 mL ddH_2_O in a 1 L glass bottle.

**CRITICAL:** Work in a flow hood and work with gloves when handling phosphoric acid. First add the water to the bottle before you add the acid to avoid an exothermic reaction and splashing of the acid.

• Then add 100 g ammonium sulfate to the 1 L glass bottle and dissolve.
• Add 20 mL of the prepared 5% (wt/vol) Serva Blue G solution.
• Mix well before each use.

### Preparation of 0.5 M EDTA solution, pH 8.0 (1 L)

**Timing: 1 h**

• Add 186.1 g disodium ethylenediamine tetraacetate 2H_2_O to 800ml of water.
• Stir vigorously on a magnetic stirrer.
• Adjust the pH to 8.0 with 2 M NaOH. EDTA will slowly go into solution as the pH approaches 8.0.
• Dispense into aliquots, and sterilize by autoclaving.
• Store at 15–25°C for up to 1 year.

**Table:** Sonication/Lysis buffer example (500 mL)

Reagent	Final concentration	Amount
urea	2 M	60.06 g
glycerol	20% (v/v)	100 mL
0.5 M EDTA solution pH 8.0	1 μM	100 μL
Triton X-100	1 %	5 mL
ddH_2_O	N/A	∼400 mL
**Total**	**N/A**	**500 mL**

Store at 15–25°C for up to 1 year.

### Preparation of sonication/lysis buffer

**Timing: 1 h**

**CRITICAL:** For every protein the optimal sonication buffer should be determined. Examples of sonication buffers that can be used are. Here a preparation protocol for two different buffers is given.

2 M urea, 20% glycerol, 1 μM EDTA, 1% Triton X-100.

• Dissolve 60.06 g urea in ∼400 mL ddH_2_O.
• Add the 100 mL glycerol to the urea solution and mix well on a magnetic stirrer.
• Add the 100 μl EDTA solution.
• Add the 5 mL Triton X-100 and mix the solution well on a magnetic stirrer.
• Fill up to 500 mL with ddH_2_O if necessary.

2 M to up to 8 M urea in PBS

• Dissolve the required amount of urea (e.g., for 2 M urea/PBS) 120.12 g in ∼800 mL PBS.
• Mix on a magnetic stirrer.
• When the urea is fully dissolved, fill up to 1 L with ddH_2_O.

### Preparation of 5 M NaCl (1 L)

• Weigh 292.20 g NaCl.
• Dissolve in ∼800 mL ddH_2_O.
• Fill up to 1 L with ddH_2_O.
• Autoclave.
• Store at 15–25°C for up to 1 year.

### Preparation of 1 M Tris, pH 8.0 (100 mL)

• Weigh 12.11 g Tris base.
• Dissolve in ∼90 mL ddH_2_O.
• Add slowly concentrated HCl with a Pasteur pipette to adjust the pH.
• Fill up to 100 mL with ddH_2_O.
• Autoclave.
• Store at 15–25°C for up to 1 year.

**Table:** 200 mM Imidazole solution (10 mL)

Reagent	Final concentration	Amount
Imidazole	13.6 mg/mL (200 mM)	136 mg
5 M NaCl solution	1 M	0.2 mL
1 M Tris solution pH 8.0	0.2 M	2 mL
ddH_2_O	N/A	7.7 mL
**Total**	**N/A**	**10 mL**

### Preparation of 200 mM imidazole solution (10 mL)

**Timing: 30 min**

**CRITICAL:** This solution should always be prepared fresh.

• Weigh 136 mg imidazole in a 15 mL Falcon tube.
• Add 0.2 mL 5 M NaCl, 2 mL 1 M Tris pH 8.0 and 7.7 mL ddH_2_O.

***Optional:*** Add 100 μL of 0.1 M PMSF solution to prevent protein degradation.

**Table:** Buffer E elution buffer (1 L)

Reagent	Final concentration	Amount
NaH_2_PO_4_·H2O	100 mM (13.8 g/L)	13.8 g
Tris base	10 mM (1.2 g/L)	1.2 g
8 M urea	480.5 g/L	480.5 g
**Total**	**N/A**	**1 L**

Aliquots can be stored at −20°C for up to 1 year.

### Preparation of buffer E elution buffer (1 L)

**Timing: 30 min**

• Dissolve NaH_2_PO_4_·H2O, Tris base in 950 mL ddH_2_O.
• Adjust pH to 4.5 with 1 M HCl.
• Fill up to 1 L with ddH_2_O.
• Prepare 15 mL aliquots and store at −20°C until use.

**Table:** 10x PBS (1L)

Reagent	Final concentration	Amount
NaCl	85 g/L	85 g
Na_2_HPO_4_·2H_2_O	17 g/L	17 g
NaH_2_PO_4_·H_2_O	2.2 g/L	2.2 g
ddH_2_O	N/A	∼895 mL
**Total**	**N/A**	**1 L**

Store at 15-25°C for up to 1 year.

### Preparation of phosphate-buffered saline (PBS), 0.1 M, pH 6.7 (1 L) (10x concentrated)

**Timing: 3 h**

• Dissolve 85 g NaCl, 17 g Na_2_HPO_4_·2H_2_O and 2.2 g NaH_2_PO_4_·H_2_O in 800 mL ddH_2_O in a 1 L glass bottle.
• Check the pH.
• If necessary, adjust the pH.
• Fill up to 1 L with ddH_2_O.
• Sterilize by autoclaving for 20 min at 15 psi (1.05 kg/cm^2^) on liquid cycle.

**CRITICAL:** Autoclave the 10× PBS if a 1xPBS dilution of this buffer is used to perform the ELISA washes.

### Preparation of PBS, 10 mM, pH 6.7 (1 L)

**Timing: 10 min**

Mix 100 mL 10× PBS with 900 mL ddH_2_O.

**Table:** Wash buffer for protein purification (500 mL)

Reagent	Final concentration	Amount
1x PBS	N/A	400 mL
5 M NaCl solution	1 M	100 mL
Tween-20 20% v/v	0.05% v/v	1.25 mL
**Total**	**N/A**	**500 mL**

Store at 15-25°C for up to 1 year.

### Preparation of wash buffer for protein purification (500 mL)

**Timing: 2 h**

• Mix 400 mL phosphate buffered saline (PBS), 100 mL 5 M NaCl, 1.25 mL 20% (vol/vol) Tween-20 in a 500 mL glass bottle.
• Place bottle on a magnetic stirrer with a magnet.
• Wait until the Tween-20 is dissolved.

**CRITICAL:** Do not add more than 0.05% Tween-20 as this washes away your bound protein.

### Preparation of 20% (vol/vol) Tween-20 (40 mL)

**Timing: 1 h**

• Add 10 mL Tween-20 to 40 mL ddH_2_O in a 50 mL tube.
• Mix on a roller bench until the Tween-20 is dissolved.
• Store at 2–8°C for up to 1 year.

**Table:** Vaccine composition Freund’s adjuvant (1.5 mL)

Reagent	Final concentration	Amount
vaccine protein (2 mg/mL)	1 mg/mL	750 μl
FCA/FIA	N/A	750 μl
**Total**	**N/A**	**1.5 mL**

**Table:** Vaccine composition Montanide/CpG adjuvant (1.5 mL)

Reagent	Final concentration	Amount
Vaccine protein (2.5 mg/mL)	1 mg/mL	600 μl
Montanide ISA 720	N/A	750 μl
CpG 1826 solution (5 mg/mL)	0.5 mg/mL	150 μl
**Total**	**N/A**	**1.5 mL**

### Antibody titer calculation

**Timing: 30 min**

**Table:** Antibody titer calculation

Parameter	Value
serum dilution a (e.g., 100, 300, 900 etc.)	OD a; First OD value >cut-off
serum dilution b (e.g., 300, 900, 2700 etc.)	OD b; First OD value <cut-off
X	(OD a-b)/(serum dilution a-b)
a – cut-off	OD a – cut-off
Z	(a – cut-off)/X
Titer (serum dilution a + Z)	–

• Calculate the statistically valid cut-off value according to Frey et al. Journal of Immunological Methods 1998.
• Determine the OD value of parameter ‘a’. This is the first OD value at a certain serum dilution above the cut-off value. Named OD a.
• Determine the OD value of parameter ‘b’. This is the first OD value at a certain serum dilution below the cut-off value. Named OD b.
• Determine the parameter ‘X’. For this the formula (OD a – OD b)/(serum dilution a – serum dilution b), which means that the difference in OD value is divided by the difference in dilution.
• Determine the parameter ‘a – cut-off’. For this the cut-off value is subtracted from the first value above the cut-off (OD a).
• Determine the parameter ‘Z’. For this ‘a – cut-off’ is divided by the parameter ‘X’.
• The titer can then be calculated from the sum of ‘serum dilution a’ and ‘Z’.

## Step-by-step method details

### Vaccine construct design

**Timing: 4 weeks**

Here we explain how our vaccine protein is constructed and designed and we describe the steps for the cloning and transformation of the plasmid into competent bacteria optimized for protein expression.

**Figure 1:**
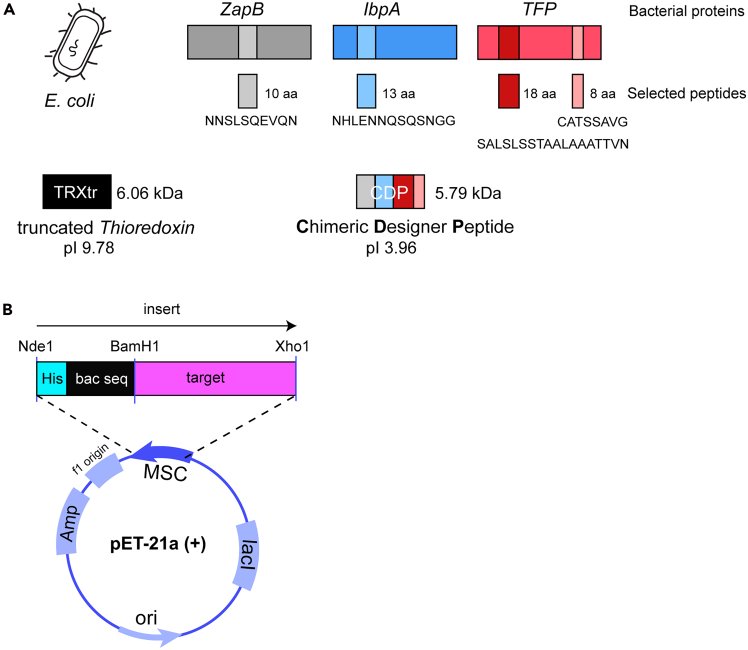
iBoost conjugate vaccine technology (A) *E. coli* protein designer sequences (carriers). Truncated thioredoxin (TRXtr) is composed of the C-terminal 58aa of bacterial thioredoxin. Chimeric designer peptide (CDP) CDP is composed of the following peptide stretches: NNSLSQEVQN (10 aa) derived from ZapB (amino acids 36–45), NHLENNQSQSNGG (13 aa) derived from IbpA (amino acids 21–33), SALSLSSTAALAAATTVN (18 aa) and GATSSAVG (8 aa) derived from TFP (amino acids 12–39 and 69–76 respectively). Adapted from.^5^ (B) Illustration of insertion of the conjugate vaccine DNA sequence into the multiple cloning site (MCS) of the pET21a plasmid. The DNA sequence is inserted between the Nde1 and Xho1 restrictions sites. The His-tag (His) is depicted in cyan, the bacterial sequence (bac seq) in black and the target in magenta. Between bac seq and target a BamH1 restriction site is placed to allow for exchange of the respective sequences.

The iBoost technology is based on the chimeric designer peptide (CDP, 66 aa) and truncated thioredoxin (TRXtr, 59 aa) (PDB: P0AA25).^1^ Truncated thioredoxin consists of the C-terminal 59 amino acids of the full length thioredoxin. CDP consists of selected clusters of amino acids with bulky hydrophilic or charged side chains, originating from three distinct bacterial (*E. coli*) proteins, the cell division protein ZapB (PDB:P0AF36), the type I fimbrial protein (A chain) (TFP, PDB: P04128) and the small heat shock protein IbpA (UniProtKB: P0C054) (Figure 1A). The selected peptide stretches are linked by one GS-linker, which are underlined in the sequence below. These conjugate domains/proteins are linked to the target antigen via a 6xGS-linker to provide flexibility to the construct. Figure 1B shows a schematic overview of the construct design including restriction sites (Nde1, BamH1 and Xho1). Between bacterial sequence and target a BamH1 restriction site is placed to allow for exchange of the respective sequences. The DNA sequence can be bought in the correct expression vector, e.g., pET21a (+) from commercial vendors such as Genscript.

Thioredoxin trunc (6.2 kDa).

MGKLTVAKLNIDQNPGTAPKYGIRGIPTLLLFKNGEVAATKVGALSKGQLKEFLDANLA (59 aa).

Chimeric Designer Peptide (5.9 kDa).

MDNNSLSQEVQNGSNHLENNQSQSNGGGSDSALSLSSKTAALAAATTVNDGSDGATSSAVG (61aa).

### Cloning

**Timing: 4 weeks**

1. Perform codon optimization of the DNA sequence of your construct (conjugate domain + target) for expression of the conjugate iBoost protein in bacteria.

***Note:*** Codon optimization of the eukaryotic DNA sequence of the target should be performed as bacteria use other tRNAs for protein expression than mammalian cells. For example, the GenSmart Codon Optimization tool (https://www.genscript.com/tools/gensmart-codon-optimization) can be used. Also, commercial vendors usually offer this service as part of their gene synthesis.

2. Clone the insert containing an N-terminal His-tag (6x His) between the restriction sites Nde1 and Xho1.

***Note:*** The His-tag can also be placed at the C-terminal of the construct.

3. Confirm the identity of the insert by Sanger sequencing using T7 promoter primers.

 T7 forward primer 5′-TAATACGACTCACTATAGGG-3′.

 T7 reverse primer 5′- GCTAGTTATTGCTCAGCGG-3′.

4. For transformation of the bacteria introduce the plasmid, range 1-10 ng, into competent *E. coli* strain BL21 DE3 or Rosetta Gami (RG) DE3 (Novagen), as these strains are optimized for protein expression.

***Note:*** BL21 DE3 is recommended for routine high-yield protein expression and proteins that fold well in the cytoplasm. The choice of RG DE3 is recommended for proteins with rare tRNA codons including human, mammalian or other eukaryotic proteins, or if expression is poor in BL21, the protein forms inclusion bodies in BL21 or requires disulfide bonds for optimal folding.

***Note:*** For plasmid propagation the *E. coli* strains DH5α or TOP10 should be used.

### Protein expression and purification

**Timing: 4 days**

Here we describe the steps required for the expression of the vaccine protein in bacteria and the purification of the protein from the bacteria to obtain a final dialyzed product, which can be used for immunization of mice.

**Figure 2:**
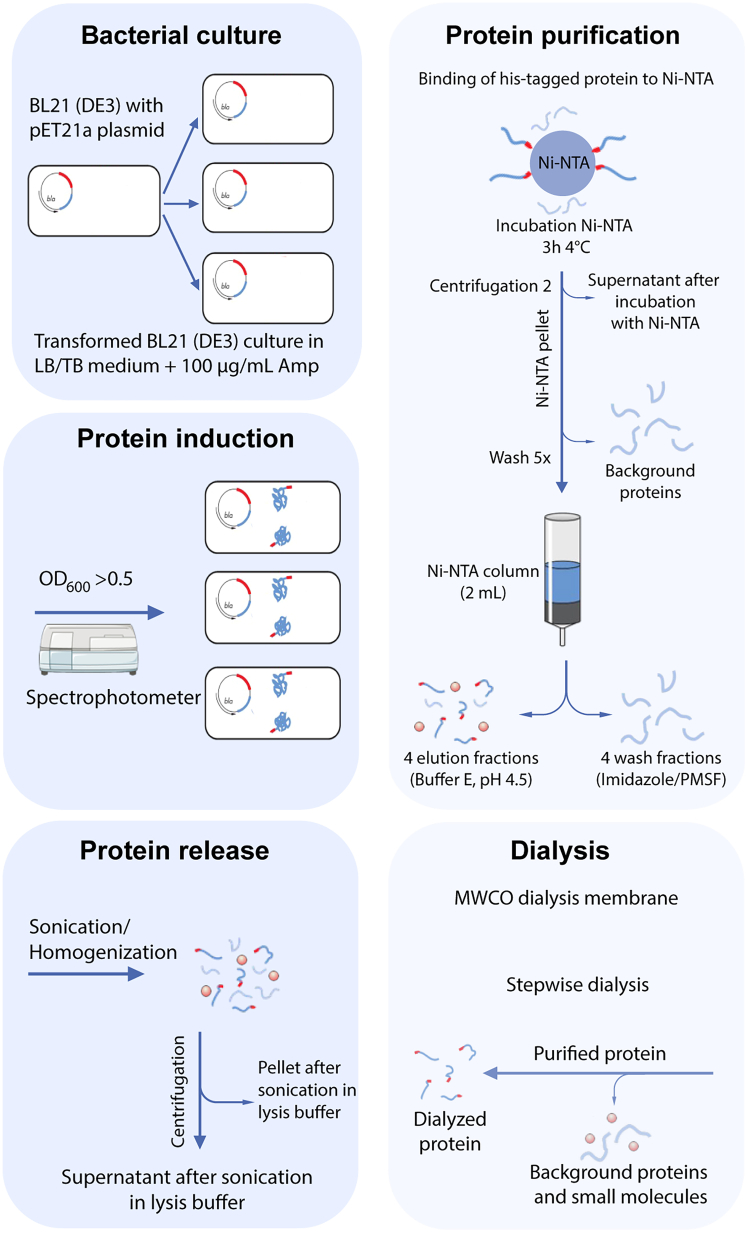
Protein expression and purification Schematic overview of the protein expression and purification process. The workflow starts with culturing of the with plasmid transformed bacteria. Next protein expression will be induced by addition of IPTG. Once the protein is produced it needs to be released from the bacteria. This is performed by e.g., sonication of the bacteria in a lysis buffer. The released protein can then be purified by IMAC, the binding to Ni-NTA agarose beads. Elution is achieved by imidazole or buffer E. Consecutive the amount of protein in the elution fractions is determined by SDS-PAGE. The fractions containing the highest amount of protein are pooled and dialyzed. (Amp; ampicillin).

### Bacterial culture

**Timing: Approximately 16 h**

***Note:*** Here a standard protein expression and purification protocol (Figure 2) is described for DNA sequences inserted into a pET21a (+) plasmid, which contains an ampicillin resistance (AmpR) gene.

5. Prepare bacteria 150 mL growth medium in a 0.5 L Erlenmeyer.

***Note:*** LB medium or TB medium containing 100 μg/mL ampicillin (Merck)

6. Prepare an overnight bacteria culture by adding a sterile pipet tip with scraped bacteria from your glycerol stock to the growth medium.
7. Put the Erlenmeyer in a laboratory shaker, 200 rpm, 37°C until the next day (∼16 h culturing time).

### Induction of protein expression in bacteria

**Timing: 4–22 h**

8. The next day, dilute the overnight culture and transfer to a 1 L Erlenmeyer.
  a. Dilute the overnight culture 1:2.6 (one part overnight culture (150 mL) and 2 parts (250 mL fresh growth medium) transfer to a 1 L Erlenmeyer. This gives an estimated optical density (OD 600 nm) of about 0.5.
  b. or dilute the overnight culture in a bigger volume of fresh growth medium and wait until the OD is at least 0.5. This usually takes 2 to 3 h and the OD has to be monitored.

***Note:*** For increased protein expression higher optical densities can be used prior to induction of protein expression. As a higher bacterial density might result in a higher protein yield. Usually, 0.5 OD is chosen to have the bacteria in the exponential growth phase in which protein expression can be induced.

9. Induce protein expression by addition of Isopropyl β-D-Thiogalactopyranoside (IPTG) to an end concentration of 1 mM.
10. Put the Erlenmeyer back into the laboratory shaker, 200 rpm, 37°C for 4 h.
  a. Or at 200 rpm, 22°C overnight (∼16 h).

***Optional:*** Protein expression can be induced at 22°C overnight (∼16h). This might enhance protein folding and subsequent purification.

***Note:*** Plasmids transformed into the bacteria should contain a *lac I* coding sequence encoding for the repressor protein, which will bind to the operator region and suppress protein expression by preventing the RNA polymerase from binding to its promoter, in the absence of lactose.

***Note:*** Protein expression can be checked by saving 5 mL of the culture prior to induction of protein expression by IPTG. Centrifuge the culture and save the bacterial pellet.

***Note:*** Optimal expression time and induction temperature should be determined for each protein. For most proteins tested 4h was optimal.

11. Centrifuge cultures at ≥4000 x g 4°C, 20 min to pellet the bacteria. Discard supernatant into an appropriate waste container.

***Note:*** It is hazardous waste and bacterial waste handling protocols should be followed.

***Note:*** 4 × 50 mL tubes should be used of which each 50 mL tube contains bacteria pellet equal to 100 mL bacteria culture in case you continue with the sonication protocol.

***Note:*** 2 × 250 mL buckets containing each 200 mL bacteria culture can be used prior to the homogenizer step.

**Pause point:** Pellets can be stored overnight at 2–8°C. However, it is recommended to freeze (−20°C) the pellets at least overnight (∼16h) before continuing. This enhances bacterial lysis and release of the protein.

### Protein release

**Timing: 3 h**

**Figure 3:**
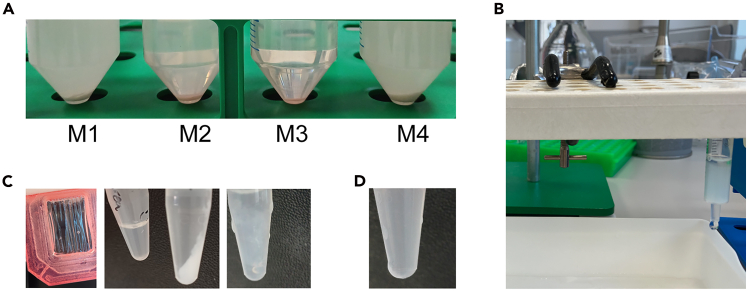
Technical details protein purification and troubleshooting (A) Examples of different sonication/lysis buffers (M1–M4) used during homogenization with the microfluidizer. The choice of buffer determines if the protein stays in solution (M2, M3) in the supernatant or precipitates (M1, M4). In M4 more protein is precipitated than in M1. In all cases pellets derived from 200 mL culture were used. M3 is also an example of the result that can be observed after sonication. (B) Example of a 2 mL syringe packed with Ni-NTA agarose beads and a glass filter in the bottom. (C) Examples of protein precipitation after dialysis. Left image: protein aggregates (visible particles) can be observed in the dialysis cassette. Middle image: in the right tube clear white protein precipitate is observed. In the left tube the protein still is in solution. Left image: a cloudy protein aggregate can be seen. This is usually observed prior to complete precipitation. (D) Protein in solution after dialysis, but the solution is saturated and slight aggregates are visible.
  b. Protocol homogenizer.
    ***Note:*** The high shear homogenizer (Microfluizider (LM10), Microfluidics) has a much higher throughput but a larger residual volume. Therefore, it recommended to grow and process a larger culture at a time. Also, prepare an excess volume of sonication/lysis buffer.
    i. Resuspend the bacterial pellet, equal to 100 mL bacteria culture, with a pipet boy in 10 mL sonication/lysis buffer.
      ***Note:*** The minimal volume that can be used in the homogenizer is 40 mL.
    ii. Prime the high shear homogenizer (Microfluizider (LM10), Mircofluidics) with sonication/lysis buffer at 2000 psi.
    iii. Homogenize minimally 3 cycles at 20 000 psi.
      ***Note:*** An optimized pressure for each protein should be chosen. Also, the number of cycles is variable and can be lowered or increased. A general protocol is presented here.
    iv. Centrifuge the homogenized pellet at ≥4000 × *g*, 4°C, 10 min to pellet debris.
    v. Pipet off the supernatant, which contains the protein, and save to a new 50 mL tube. Also, save the pellet.
      ***Note:*** Depending on the sonication buffer used there is a risk that you will pellet the protein/spin it down by centrifugation (Figure 3A). If the pellet is large and whitish then you should continue with the pellet for purification instead of the supernatant.
      **Pause point:** Freeze supernatant/pellet overnight (∼16 h) or for up to 1 month at −20°C before proceeding with the protocol.

12. Prepare the sonication/lysis buffer.
  ***Note:*** The sonication/lysis buffer should be determined for each protein. Examples of sonication buffers that can be used are:
  a. PBS.
  b. 2 M to up to 8 M urea in PBS.
  c. 2 M urea, 20% glycerol, 1 μM EDTA, 1% Triton X-100.
13. Meanwhile, thaw the bacteria pellets at 15-25°C on the bench.
  ***Note:*** In case a sonicator is used, it should be strong enough to lyse the bacteria. From own experience not every sonicator works. Recommended is the Soniprep MSE or the use of a high shear homogenizer. A homogenizer destroys the bacterial membranes by high shear force.
  a. Protocol **sonicator.**
    i. Resuspend the bacterial pellet with a pipet boy and a 10 mL pipet in the sonication buffer. 5 mL sonication buffer per 50 mL original culture used in step 4.
      **CRITICAL:** A maximum volume of 12.5 mL can be used for sonication at a time using the Soniprep MSE. This implies pellet from 100 mL bacteria culture is dissolved in 10 mL sonication buffer. A larger volume will impair the sonication and make bacteria lysis inefficient.
    ii. Sonicate the solution on ice 15 cycles of 20s ‘on’ and 30s ‘off’, Amplitude 22–24 microns, Soniprep 150 MSE.
      ***Note:*** The number of cycles, the cycle ‘on’ and ‘off’ time and the amplitude can be varied to optimize protein release, e.g. 12 cycles of 30s ‘on’ and 30s ‘off’.
    iii. Add immediately after sonication 1mM phenylmethylsulfonyl fluoride (PMSF) to prevent protein degradation.
      **CRITICAL:** Do not add the PMSF prior to sonication because this will prevent binding of the protein to the Ni-NTA agarose.
    iv. Centrifuge the sonicated pellet at ≥4000 × *g*, 4°C, 10 min to pellet debris.
    v. Transfer the supernatant, which contains the protein, to a new 50 mL tube. Also, save the pellet.
      ***Note:*** Depending on the sonication buffer used there is a risk that you will pellet the protein/spin it down by centrifugation (Figure 3A). If the pellet is large and whitish then you should continue with the pellet for protein purification instead of the supernatant.
      **Pause point:** Freeze supernatant overnight (∼16 h) or for up to 1 month at −20°C before proceeding with the protocol. Troubleshooting 1.
      **CRITICAL:** Freezing of the supernatant or pellet is critical, as this will enhance purification.

### Protein purification

**Timing: 8**–**21 h**

14. Thaw supernatant/pellet at 15–25°C on the bench.
15. Add 300 μL/500 μL nickel-nitrilotriacetic acid (Ni-NTA) agarose beads to 10 mL supernatant (equal to 100 mL bacteria culture) to the 50 mL tube.

**CRITICAL:** The lowest amount Ni-NTA agarose that can be used is 200 μL, which is equal to 100 μL packed beads in the column.

***Note:*** More supernatant can be used to enhance purification (concentrate), as the Ni-NTA agarose has overcapacity. Depending on the amount of the protein in the supernatant the amount of Ni-NTA agarose beads should be adjusted.

***Note:*** When continuing with the pellet; dissolve the pellet in 5–10 mL sonication/lysis buffer per 100 mL bacteria culture and then add the Ni-NTA agarose. Troubleshooting 2.

16. Incubate the 50 mL tube on a roller bench in the cold room at 2–8°C for 3 h or overnight (∼16 h).

***Note:*** Overnight (∼16 h) incubation is recommended. However, sufficient protein can be bound to the Ni-NTA agarose as well by binding at room temperature (15–25°C) for 2–2.5 h on a roller bench. Also, some proteins might require binding at room temperature (15–25°C).

***Optional:*** Add 20 mM imidazole during Ni-NTA agarose binding to reduce binding of background proteins to the Ni-agarose.

17. Spin down the Ni-NTA agarose beads at 1500 × *g*, 5 min, 4°C.

**CRITICAL:** Save supernatant to be able to use it in a second purification round and to check if all protein has bound to the Ni-NTA agarose beads.

18. Wash the Ni-NTA agarose beads 5× with 25 mL wash buffer. Spin down the Ni-NTA agarose at 800 × *g*, 3 min, 4°C. Discard wash buffer after each centrifugation step and replace with new wash buffer.
  a. Wash buffer: 1 M NaCl, PBS, 0.05% Tween-20.

***Note:*** The amount of NaCl and Tween-20 can be adjusted to remove more background proteins.

**CRITICAL:** Do not use a too high amount of Tween-20, because this will remove your bound protein from the beads.

19. Wash the Ni-NTA agarose 1 time with 25 mL PBS. Spin down the Ni-NTA agarose at 800 × *g*, 3 min, 4°C. Discard the supernatant.
20. Dissolve/resuspend the Ni-NTA beads in fresh PBS, approx. 1– 5 mL.
21. Prepare a column, by adding a glass filter (Sartorius) in the bottom of a 1 or 2 mL syringe (Figure 3B).

***Note:*** Depending on the amount of Ni-NTA agarose beads and the amount of bound protein the syringe should be chosen accordingly.

22. Transfer the resuspended Ni-NTA beads to a column with a pipette.
23. Let the resin settle and the liquid flow-through.
  **CRITICAL:** Do not let the column stand dry.
  ***Optional:*** Pre-wash column with 4 column volumes (volume of the packed beads, which is half the volume of Ni-NTA agarose used) of 20 mM and or max. 40 mM imidazole to remove background binding.
  ***Note:*** Higher imidazole concentrations will also elute your protein. This sometimes already happens at a concentration of 40 mM.
  ***Optional:*** In case removal of lipopolysaccharide (LPS)/endotoxin is desired this can be performed with Triton X-114 at 2–8°C.^6^
  a. Prepare the PBS/0.1% Triton X-114 solution one day prior to use. Put into the refrigerator (4°C) to dissolve the Triton X-114.
  b. Wash the Ni-agarose on the column with 50 (at least 20) column volumes of PBS/0.1% Triton X-114 in the cold room at 2–8°C.
  c. Next, wash the column with 20 (at least 5) column volumes of PBS in the cold room at 2-8°C to remove the Triton X-114.
  d. Continue with elution of the protein at room temperature (15–25°C).
24. Elute bound protein with 4 fractions (150 μL/250 μL equal to one column volume, the amount of packed beads, which is half the volume of Ni-NTA agarose used) 200 mM imidazole solution. Catch separate fractions in a 1.5 mL Eppendorf tube.
  a. 200 mM imidazole solution: 200 mM Imidazole, 1 M NaCl, 200 mM Tris pH 8.0, 1 mM PMSF.
    ***Note:*** The PMSF might be omitted if your protein is stable.
    ***Note:*** Always prepare the imidazole solution fresh. Troubleshooting 3.
    ***Optional:*** If the protein does not elute with imidazole use buffer E solution or EDTA instead.
  b. Buffer E: 100 mM NaH_2_PO_4_, 10 mM Tris, 8 M urea, pH 4.5.
  c. EDTA: 50 – 500 mM EDTA.
    ***Note:*** EDTA strips the nickel of the agarose beads. It might interfere with the solubility of your protein as it captures divalent cations as well as downstream applications.
    **Pause point:** Eluted fractions can be stored overnight (∼16 h) at 2-8°C or frozen at −20°C until the SDS-PAGE can be performed.

### SDS-PAGE

**Timing: 8**–**19 h**

25.Determine the protein amount per fraction by SDS-PAGE and Coomassie staining.

**Figure 4:**
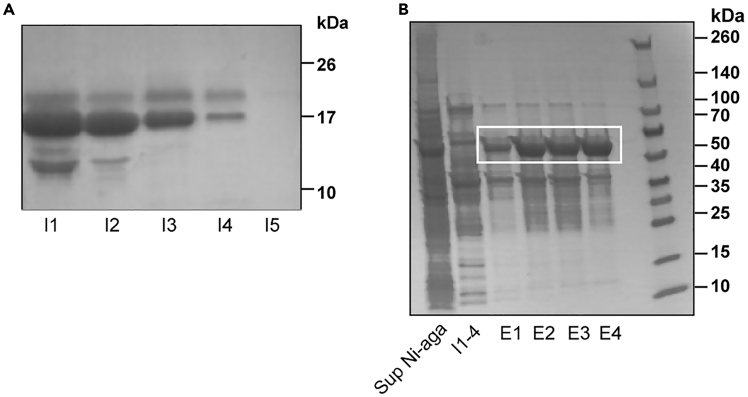
Expected outcomes of the protein purification (A) Purified murine TRXtr-mCD99 protein (18 kDa). Imidazole elution fractions (I) are shown. Adapted from.^2^ (B) Purification of murine TRXtr-mVim (61 kDa). The TRXtr-mVim protein is released into the supernatant after sonication. The left lane shows the supernatant after binding of the protein to Ni-NTA agarose (Sup Ni-aga). The protein cannot be eluted with imidazole (I1-4) but is eluted with buffer E (E1–E4) (white box).

***Note:*** Include on your gel also the supernatant and the pellet after sonication/homogenization and the supernatant after Ni-NTA agarose. See Figure 4 for an example of the gel after Coomassie staining.

26. Add per sample: SDS sample buffer (2×) 10 μL, sample 10 μL, sample reducing agent (10×) 1μL.

***Note:*** Dilute the pellet 5× before loading onto the gel to prevent protein overload.

***Note:*** The used wash buffer can also be included on the gel to check if the protein is not lost during the washing step.

27. Incubate samples 10 min at 95°C in a heating block.
28. Load 21 μL sample per well in a Novex™ Tris-Glycine Mini Protein Gel.

***Note:*** These volumes can be both run on 10 well or 15 well gels.

29. Prepare 1× Tris-Glycine SDS running buffer.
30. Run the gel at 200 V for approx. 40 min in the Mini Gel Tank.
31. Stain the gel with Coomassie stain solution (4 parts colloidal Coomassie solution + one part methanol) ≥3 h or overnight (∼16 h) at 15–25°C on a rocker at 30 rpm.
32. Destain for 1 min with methanol and then further with ddH_2_O on a rocker.
33. Refresh the ddH_2_O every hour.

***Note:*** Minimal destaining time is 3 h.

***Note:*** If a lot of protein remains in the supernatant after the first purification step, then another purification step can be performed on the same supernatant. Up to 3 consecutive purification rounds can be performed. In between purifications; supernatants can be frozen at −20°C.

34. Confirm protein identity by western blot and or mass spectrometry.

### Dialysis

**Timing: 8**–**23 h**

35. Determine from the SDS-PAGE gel which fractions can be pooled prior to dialysis.

***Note:*** Pool fractions with similar protein content. Pooling all fractions will dilute your protein too much and you will have difficulties concentrating it afterwards. In case your protein content is high, you might want to dilute your pooled fractions in max. 1:1 in elution buffer (200 mM imidazole or buffer E) prior to dialysis.

36. Transfer the sample to a Silde-A-Lyzer G3 dialysis cassette with an appropriate container volume and molecular weight cut-off (MWCO) and place into a beaker glass with a volume of at least 500 mL dialysis buffer and a stirring magnet.
37. Put the beaker glass on a magnetic stirrer at low speed in the cold room and dialyze overnight (∼16 h).
38. The next day exchange the dialysis buffer every 2 h, in consecutive steps until the desired buffer is reached and the protein does not precipitate in the solution (Figures 3C and 3D).

***Note:*** For every protein the desired dialysis buffer and protocol need to be identified. Troubleshooting 4.

39. Determine protein concentration/amount by BCA protein assay.

**Pause point:** The dialyzed protein can be stored at −20°C for up to 1 year or at 2–8°C for 2 weeks.

### Vaccine testing in mice

**Timing: 8 weeks**

Here we describe the steps to be able to test the generated vaccine, the in *E. coli* produced and purified protein plus adjuvant, in mice. In addition, a protocol is given to evaluate the antibody response (humoral immune response) induced in the mice by ELISA.

***Note:*** Mouse experiments should always be performed considering animal welfare according to national rules and regulations. Prior to execution of the mouse experiments an animal license from the national and local animal welfare bodies should be obtained. Additionally, personnel must be trained.

**CRITICAL:** For immunization against a self-antigen (e.g., a tumor vascular antigen),^3^^,^^7^ the immunological tolerance needs to be circumvented, therefore at least 3–4 vaccinations are required to obtain sufficient antibody titers. Immunization against a foreign antigen requires 1–2 vaccinations only.^5^

### Vaccine preparation

**Timing: 1 h**

***Note:*** Volumes required for preparation of the vaccine emulsion are listed in the materials and equipment section.

***Note:*** The injection volume of the vaccine is 100 μL per mouse. The vaccine protein dose is 100 μg per mouse.

***Note:*** For induction of an immune response against a self-antigen a potent adjuvant such as Freund’s adjuvant or Montanide ISA 720/CpG1826 is required to break the immunological self-tolerance.^4^

***Note:*** In case Freund’s adjuvant is used; primer vaccinations are given with Freund’s complete adjuvant (FCA), which contains heat inactivated *Mycobacterium tuberculosis*, followed by booster vaccinations with Freund’s incomplete adjuvant (FIA).

***Note:*** The Montanide ISA 720 (MN720) adjuvant (Seppic) is always used in combination with the CpG oligonucleotide 1826; CpG dose 50 μg per mouse.^4^

***Note:*** For induction of a humoral immune response against a poorly immunogenic foreign antigen the Sepivac adjuvant (Seppic) can be used.^5^^,^^8^ In this case, a foreign protein is targeted, the injected protein dose per mouse might be lowered, e.g. to 30 μg, 10 μg or 3 μg^7^. The dose however should be titrated. In addition, immunization against a foreign antigen requires fewer vaccinations than against a self-antigen, as the immunological tolerance does not need to be broken.^5^

***Note:*** Vaccine emulsions are prepared as a batch for a maximum of 10 mice at a time. 50% extra volume is prepared to account for loss in the syringes and extractable volume from the Eppendorf tube.

40. Pipet the in the table listed volumes (materials and equipment section) into a 2 mL Eppendorf tube.
41. Seal the lid with parafilm.
42. Place the Eppendorf tube onto a Vortex Genie 2 with an adapter for Eppendorf tubes.

**CRITICAL:** The type of vortex used here is important. It is recommended to use the Vortex Genie 2 as other vortexes are not potent enough to obtain a good emulsion.

43. Vortex samples at full speed for 30 min at 15–25°C to obtain a vaccine emulsion.
44. Spin down the samples in a microcentrifuge for ∼20s.

***Note:*** Avoid centrifuging for a long time as separation of the emulsion may occur.

**CRITICAL:** Inject vaccine emulsion prepared with Freund’s adjuvant immediately after preparation to avoid too high viscosity for injection.

### Vaccination of mice

**Timing: 1**–**3 h**

***Note:*** Preferably the vaccine emulsion should be injected into the mice within a time frame of 2 h after preparation. It is advised to prepare the vaccine emulsions in the animal facility.

45. Place a blue needle (23Gauge) onto the syringe. Suck up the vaccine emulsion into a 1 mL luer-lock syringe (ref 309628, BD).
46. Avoid aspirating of air. To this end aspirate first part of the emulsion, e.g., until half the syringe is filled. Eject the volume again and slowly suck up the emulsion again.

**CRITICAL:** Use a luer-lock syringe as the vaccine emulsion is very viscous. Otherwise, the needle might loosen from the syringe, or the plunger might break during aspiration of the emulsion into the syringe.

**CRITICAL:** Change the needle on the syringe to an orange needle (25Gauge) prior to injection.

47. Restrain the mouse and slowly inject 100 μL subcutaneously into the left groin.

***Note:*** Booster vaccinations should always be administered in the same groin, to activate the same lymph nodes.^9^

***Note:*** To avoid spill of vaccine emulsion, 10 mice should be vaccinated consecutively with the same needle. Only if the needle gets blunt change it.

48. The next day weigh the mice and check the groin of the mice for possible occurrence of ulceration.

***Note:*** Mice should be checked regularly during the experiment for presence of ulcers at the injection site.

### Blood sampling

**Timing: 2**–**4 h per sampling round (depending on the number of mice)**

49. Take a blood sample prior to start of the study, which will be the null serum.
50. Thereafter, take blood samples one week after each vaccination.
  ***Note:*** A maximum volume of 100 μL blood per mouse is taken per blood sample.
  a. Blood sampling from the tail vein:
    i. The mouse is warmed up in a Thermacage (39–40°C) for about 5 min.
      ***Note:*** A lower temperature might be chosen, e.g. 38°C to prevent overheating of the mouse. Also, the warming up time might be shorter than 5 min.
    ii. Make a small cut in the tail vein with a scalpel.
    iii. Collect the blood in an Eppendorf tube.
    iv. Stop bleeding by applying gentle pressure to the tail for one minute.
    v. Place the mice in a separate cage for a while to avoid contamination of the housing cage.
  b. Blood sampling from the vena saphena:
    ***Note:*** Using this method the mouse does not need to be warmed up prior to blood collection.
    i. Fix the mouse in a hairnet.
    ii. Lay it on its flank.
    iii. Shave the thigh.
    iv. Fix the hindleg by pushing up. In the middle of the leg the vena saphena is located. By compressing the vein in the groin the vena saphena will be congested and better visible.
    v. Punch the vena saphena with an orange needle (25 Gauge).
    vi. Collect the blood via a microcapillary into a blood collection tube. Alternatively, a microvette collection tube can be used, as it contains a microcapillary inside.
      **CRITICAL:** Use collection tubes without heparin, as you want to collect the serum.
51. Let the blood coagulate overnight (∼16 h) at 2-8°C in the fridge.
52. The next day centrifuge the tubes at max. 4500 *g*, 10 min, 4°C in a microcentrifuge.
53. Collect the supernatant (serum) into a new 1.5 mL Eppendorf tube.
54. Centrifuge the tubes with the supernatant again.
55. Collect the supernatant to a new 0.5 or 1.5 mL Eppendorf tube.

**Pause point:** Store the serum at −20°C until use.

### ELISA: Antibody read-out

**Timing: 2 days**

***Note:*** In case available recombinant proteins for the targets to which the vaccine is directed can be purchased from a commercial vendor and used for coating of the ELISA plates. However, if purchase is not an option the protein, lacking the conjugate domain, can be produced in house. In case the protein for coating is produced in house, the sera should be diluted in Rosetta gami or BL21 bacteria extract to block background binding in the ELISA.

### Preparation of Rosetta-gami/BL21 bacterial extract

**Timing: 17 h**

***Note:*** Rosetta gami DE3 and BL21 extract for use in ELISA is produced from uninduced pET21a (+)-TRX transformed overnight cultures. The pET21a (+)-TRX vector map is included in the supplementary material.

***Note:*** Bacteria containing the plasmid encoding the foreign conjugate of the vaccine protein should be used.

56. Prepare 400 mL overnight cultures as described in Protein expression and purification.
57. The next day divide the bacteria culture over 4×50 mL tubes, to obtain bacterial pellet from 100 mL culture per 50 mL tube.

***Note:*** Perform steps 57 to 59 twice.

58. Centrifuge at 4000 *g*, 10min, 4°C (Rotina 420 R, Hettich).
59. Discard the culture medium in an appropriate container.

***Note:*** Discard according to national rules and regulations.

***Note:*** It is hazardous waste due to use of genetically modified bacteria and antibiotics in the culture medium.

60. Wash the pellet 3 times with PBS.
  a. Resuspend the pellet in 10 mL PBS first with a pipet boy.
  b. Then fill up with PBS to 25 mL.
  c. Centrifuge at ≥4000 *g*, 10min, 4°C.
  d. Discard the supernatant and repeat step a-d 2 times.
61. Dissolve the bacterial pellet originating from 100 mL culture in 10 mL PBS.

Sonicator: Sonicate 15 cycles 20s ‘on’ and 30s ‘off’ (amplitude 22– 26 microns, Soniprep 150 MSE).

Homogenizer: Process 200 mL dissolved pellet 3 rounds of 10000 psi and 1 round of 20000 psi.

***Note:*** In the homogenizer larger volumes can be processed. Also, a larger volume is needed as some of the volume will be lost during processing. For optimal result at least a volume of 150 mL should be used. This means that more or bigger cultures should be prepared.

62. Centrifuge the bacterial lysates at ≥4000 *g*, 20min, 4°C.
63. Transfer the supernatants to a new 50 mL tube, which is your final RG/BL21 extract.

**Pause point:** Store supernatants at −20°C until use.

### ELISA

**Timing: 6**–**24 h**

***Note:*** For detection of antibodies in the sera of the mice the ELISA should be optimized for each target.

***Note:*** Use 50 μL per ELISA well, unless otherwise indicated.

64. Coat 96-well ELISA plates with target protein.

***Note:*** Coating protein concentrations are usually between 2 μg/mL and 8 μg/mL in an appropriate coating buffer (e.g. PBS or 0.5M urea in PBS). The coating buffer is dependent on the formulation buffer into which the protein was dialyzed.

65. Cover the plates with Easy seal and incubate for 1 h at 37°C or at 4°C for ∼16 h (overnight).
66. Discard the volume in the wells.
67. Block the ELISA plate with 4% milk in PBS. 100 μL blocking solution per well for 1 h at 37°C.

***Note:*** The blocking solution can be adjusted accordingly to what works best. Other options are horse serum or 5% BSA.

**CRITICAL:** Centrifuge the horse serum at ≥4000 g, 10 min, 4°C in a 50 mL tube to remove debris prior to use in ELISA.

68. Discard the volume in the wells.
69. Wash 1 min in PBS. Discard the volume in the wells.

***Note:*** A spray flask can be used, a multi-channel pipette or a cap washer. Make sure that the wells are properly filled with washing buffer.

70. During the blocking step dilute the mouse sera 1:10 in horse serum. Dilute the sera further 1:10 in 10% Rosetta Gami extract/BL21 extract in PBS to obtain a final serum dilution of 1:100.

***Note:*** Mouse sera can also be diluted in 4% milk instead of horse serum.

***Optional:*** The amount of bacterial extract can be increased to reduce non-specific binding of the sera. Up to 100% bacterial extract can be used. Troubleshooting 5.

71. Incubate the plate for 45 min at 37°C with serum.
72. Discard the volume in the wells.
73. Wash 1 min, 5 min, 1 min, 5 min with PBS. Discard the volume in the wells between each wash.

***Optional:*** Alternatively, the plate can be washed with PBS-Tween 20 (T) 0.01% to remove non-specific background.

74. Incubate plate with biotinylated polyclonal goat anti-mouse Ig (E0433, Dako Cytomation), diluted 1:2000 in PBS-T 0.01% for 45 min at 37°C.
75. Wash 1 min, 5 min, 1 min, 5 min with PBS. Discard the volume in the wells between each wash.
76. Incubate plate with streptavidin-HRP (P0397, Dako Cytomation), diluted 1:2000 in PBS-T 0.01% for 30 min at 37°C.
77. Wash 1 min, 5 min, 1 min, 5 min with PBS. Discard the volume in the wells between each wash.
78. Add TMB substrate and incubate 5–15 min at 15–25°C.
79. Read absorbance at 655 nm with a microplate reader.
  a. Antibody titer measurement.
    i. Dilute the sera as described in step 72 and then further in dilution steps of three, e.g., 1:100, 1:300, 1:900, 1:2700, 1:8100, 1:24300.
      ***Note:*** Data can be presented as individual dilution curves, or the antibody titer can be calculated according to Frey et al.^10^ and the protocol in the materials and equipment section.
  b. Antibody isotype measurement.
    ***Note:*** Antibody isotypes are measured according to the outlined ELISA including the following adjustments: sera are only tested in a 1:100 and 1:1000 dilution.
    ***Note:*** The following secondary antibodies are used in (step 76) in a 1:2500 dilution: biotinylated polyclonal goat anti-mouse IgG1 (Southern Biotech, Cat. 1070-08), IgG2a (Southern Biotech, Cat. 1080-08). IgG2b (Southern Biotech, Cat. 1090-08), IgG2c (Southern Biotech, Cat. 1079-05), IgG3 (Southern Biotech, Cat. 1100-08) and IgM (Southern Biotech, Cat. 1020-08).

## Expected outcomes

Proteins that bind via their 6xHis-tag can be eluted with imidazole (I) (Figure 4A). Proteins that bind on overall charge (pI) will not elute by adding imidazole (Figure 4B). TRXtr-mVim for example is not eluted in the pooled imidazole fractions (I1-4). This protein is eluted when the pH of the elution buffer is lowered to pH 4.5, fractions E1-E4. This procedure protonates the protein and thus will repel it from the Ni-NTA agarose. Frequently, the protein is eluted in the second imidazole fraction or in the buffer E fractions (pH<4.5) (Figure 4B). Often still protein is left in the supernatant after incubation with Ni-NTA agarose (Figure 4B, sup Ni-aga). The supernatant can then be reused to purify additional protein. This can be done up to three purification rounds. It also can be the case that in the first purification round (incubation with Ni-NTA agarose plus elution with imidazole and/or buffer E) no protein is released. Then new Ni-NTA agarose beads can be added to the supernatant and the protein can be purified in a second round.

**Figure 5:**
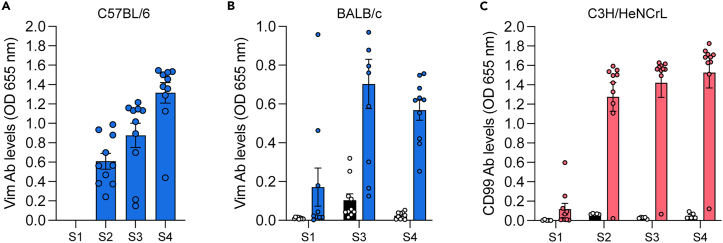
Expected outcomes antibody levels (A) Antibodies induced against the self-antigen vimentin after vaccination with TRXtr-mVim in C57BL/6 mice. Error bars represent ± SEM. Adapted from.^3^ (B) Antibodies against vimentin induced after vaccination with TRXtr-mVim (blue bars) in BALB/c mice. Control vaccinated mice are devoid of antibodies against vimentin (black bars). Error bars represent ± SEM. Adapted from.^3^ (C) Antibodies against CD99 induced after vaccination with TRXtr-mCD99 (pink bars) in C3H mice. Control vaccinated mice are devoid of antibodies against CD99 (black bars).Error bars represent ± SEM. Adapted from.^2^

When vaccinating against a self-antigen e.g., CD99, or vimentin, it can be anticipated that after the first vaccination (S1) (Figures 5A and 5C) no antibodies are measured in the sera of the mice.^2^^,^^3^^,^^4^ After the second vaccination (S2) (Figures 5A and 5C) antibodies are coming up. After vaccination against a foreign antigen, e.g., the receptor binding domain (RBD) of SARS-CoV2, high antibody titers were detected after only two vaccinations.^5^ For most self-antigens antibody titers can be further increased by administering a 4^th^ vaccination (Figures 5A and 5C).

## Limitations

While the iBoost technology represents a significant advancement in breaking immune tolerance to self-antigens, several considerations remain for its broader clinical application. Although the bacterial carrier sequences are specifically engineered to be small and moderately immunogenic, there remains a possibility that in some settings, the immune response may partially skew toward the carrier. Ongoing optimization of carrier-target ratios and linker design may help mitigate this risk.

As with most vaccine platforms, the strength and specificity of the antibody response can vary between individuals due to differences in immune status, HLA genotype, and other host factors. Personalized approaches or target selection strategies may enhance consistency across patient populations.

As expected for vaccines targeting self-antigens, multiple doses are typically required to achieve and sustain high antibody titers. This is due to the need to overcome immunological tolerance. While repeated dosing introduces some logistical complexity, protocols using long-acting adjuvants or alternative delivery platforms (e.g., slow-release formulations) may reduce the number of required injections over time.

Because iBoost aims to break tolerance, theoretical concerns regarding autoimmunity exist. This is dependent on the selected target, but extensive preclinical experience against many targets has shown that this does not lead to major adverse autoimmune manifestations.^2^^,^^3^ To further minimize risk, target selection can prioritize antigens with restricted expression in diseased tissues or tumor vasculature, and safety can be rigorously monitored in clinical trials.

For tumors with low target antigen expression, antibody-mediated effects may be limited. In such cases, combining iBoost vaccines with synergistic therapies, such as immune checkpoint inhibitors or agents that upregulate antigen expression, may enhance therapeutic efficacy.

In general, bacterial expression has a protein size limitation, and proteins with a size larger than 80 kDa are difficult to produce or cannot be produced at all. This limitation can be overcome by choosing plant- or eukaryotic expression systems of e.g., insect- or mammalian cells. However, these systems perform protein glycosylation, which might be unwanted in some cases.

Although iBoost constructs are well suited to recombinant expression in *E. coli*, translation to clinical-grade (GMP) production requires optimization of purification protocols, endotoxin removal, and quality control systems. These are standard steps in biologic development and can be addressed through established manufacturing pipelines.

Finally, the iBoost platform relies on potent adjuvants, such as Montanide or Sepivac, to drive robust immune responses. While some formulations may cause local or systemic reactions, these effects are generally manageable. Alternative adjuvants or formulation improvements, such as reduced-volume emulsions or novel toll-like receptor (TLR) agonist combinations, may further improve tolerability without compromising efficacy.

Taken together, these considerations highlight areas for refinement, but none represent insurmountable barriers. With continued development, iBoost offers a practical, scalable, and adaptable platform for therapeutic vaccination against self- and low-immunogenic antigens.

## Troubleshooting

### Problem 1: Protein release (step 13a)

The protein is not released into the supernatant (Figure 3A). The sonication buffer might be unsuitable.

### Potential solution

For every protein the optimal sonication buffer should be determined. Examples are given in the materials and equipment section.

### Problem 2: Protein purification (step 15)

The protein does not bind well to the Ni-agarose.

The protein yield is low after Ni-agarose purification.

### Potential solution

• Determine the isoelectric point (pI) of the protein on the Expasy website (www.expasy.org), under Compute pI/MW. Change the pH of the protein solution to two pH units higher than the pI of the protein. This will give the protein a net negative charge and therefore enhances its binding to Ni-agarose. Note that the pH of the protein solution should not be higher than pH 9. Above this pH the Ni-agarose will not work anymore.
• Dissolve the bacterial pellet in a smaller volume of sonication buffer instead (5 mL instead of 10 mL) to concentrate the protein and increase Ni-agarose binding.

### Problem 3: Protein purification (step 24)

The protein does not elute with imidazole only.

### Potential solution

• 8 M urea can be added to the imidazole solution.
• Also, if protein binding is very strong the column can first be washed with 4 fractions of 200 mM imidazole solution before elution with buffer E. By lowering the pH to 4.5 the protein mostly can be eluted on its charge.
• In case elution with buffer E cannot be achieved the Ni-agarose can be stripped from the beads with EDTA.
• In case the protein can be eluted with imidazole solution, but a lot of background is seen, the column can be washed with fractions of a low concentration of imidazole (e.g., 4x 20 mM imidazole, followed by 4x 40 mM imidazole). However, there is a risk that also part of the desired protein will elute at these imidazole concentrations. Then lower concentrations of imidazole can be tried.

### Problem 4: Dialysis (step 38)

Protein precipitation occurs during the dialysis step (Figure 3C).

### Potential solution

• Determine from the SDS-PAGE gel which samples can be pooled. It is advised to pool fractions with a similar protein content to avoid dilution of the sample, as concentration afterwards might be difficult. In case the protein content of each fraction is very high the pooled protein fractions can be diluted in dialysis buffer prior to dialysis. This might prevent precipitation/aggregation during dialysis.
• For each protein the optimal dialysis buffer should be determined.

 Examples of dialysis buffers:

 PBS.

 Urea/PBS.

 Urea/2 mM phosphate buffer pH 7.5.

• If PBS is used the protein can be dialyzed overnight in a dialysis cassette placed into a beaker glass with at least 100x the volume of the protein to be dialyzed as dialysis solution. Place on a magnetic stirrer in the cold room (2-8°C) and dialyze overnight (∼16 h). The next day replace the PBS and dialyze for 2 h at 2-8°C. If a buffer with urea is used, perform e.g., stepwise dialysis overnight (∼16 h) in 4 M urea buffer, followed by 3 M urea (2 h), 2.5 M urea (2 h) and 2 M urea (2 h) buffer. Depending on the protein the urea concentration might be lowered further stepwise.

### Problem 5: ELISA (step 70)

Non-specific binding is observed in the ELISA.

### Potential solution

• The amount of bacterial extract can be increased to reduce non-specific binding of the sera. Up to 100% bacterial extract can be used.
• The bacterial extract can be derived from *E. coli* Rosetta Gami DE3 or *E. coli* BL21 DE3. Especially in our ELISA for the determination of anti-vimentin antibodies we observed significant background binding of the vimentin antibody negative control sera. To solve this problem, we diluted the sera 1:10 in 0.2 mg/mL TRX protein in PBS. Purification of TRX is described in van Beijnum et al.^3^

## Resource availability

### Lead contact

Further information and requests for resources and reagents should be directed to and will be fulfilled by the lead contact, Arjan W. Griffioen (a.griffioen@amsterdamumc.nl).

### Technical contact

Technical questions on executing this protocol should be directed to and will be answered by the technical contact, Elisabeth J.M. Huijbers (e.huijbers@amsterdamumc.nl or ejm.huijbers@cimcure.com).

### Materials availability

Neither materials nor any new unique reagents were generated in this study.

### Data and code availability

All data presented are derived from the experiments reported in Huijbers et al., Van Beijnum et al., and Van Loon et al.^1^^,^^2^^,^^3^^,^^4^

## Acknowledgments

We thank Emma Bos, Quinty Hansen, Pien Maarschalkerweerd, Nadia Hammi, Emilie Roth, Emiline Barras, Giulia Bressan, Irene Querol Velilla, Anton Yang, Celine van der Hulst, and Yuan Pétermann for technical assistance.

This work was supported by the EU, FP7-PEOPLE-2012-IEF, GENE, ID: 328695 (E.J.H.); the 10.13039/501100004622Dutch Cancer Society KWF 2018-11651 (E.J.H. and A.W.G.); and the 10.13039/100016036Health∼Holland, PPP Allowance LSHM18007 (A.W.G).

## Author contributions

Conceptualization, E.J.M.H., J.R.v.B., and A.W.G.; methodology, E.J.M.H., A.W.G., J.R.v.B., J.K., G.E.W., P.C.J.K., L.M., S.M., K.v.L., and M.A.C.; investigation, E.J.M.H., J.K., G.E.W., P.C.J.K., L.M., S.M., K.v.L., and M.A.C.; visualization, E.J.M.H.; funding acquisition, E.J.M.H. and A.W.G.; project administration, E.J.M.H., J.R.v.B., and A.W.G.; supervision, E.J.M.H., J.R.v.B., and A.W.G.; writing – original draft, E.J.M.H.; writing – review and editing, E.J.M.H., J.R.v.B., A.W.G., J.K., G.E.W., P.C.J.K., L.M., S.M., K.v.L., and M.A.C.

## Declaration of interests

E.J.M.H. and A.W.G. are inventors on 2 patents filed for the iBoost technology: PCT/NL2017/050526 (US20230340045A1), “Embryonic angiogenesis markers and diagnostic and therapeutic strategies based thereon” (inventors: A.W.G., E.J.M.H., and Patrycja Nowak-Sliwinska); and PCT/NL2023/050226, “Novel peptide conjugate vaccines” (inventors: A.W.G., E.J.M.H., and H.v.L.). A.W.G. holds shares in CimCure. E.J.M.H. is employed full-time and A.W.G. part-time by CimCure BV.

## Supplemental information

Data S1. Vector map TRX-His protein

## References

[1] Huijbers E.J.M., van Beijnum J.R., Lê C.T., Langman S., Nowak-Sliwinska P., Mayo K.H., Griffioen A.W. (2018). An improved conjugate vaccine technology; induction of antibody responses to the tumor vasculature. Vaccine.

[2] Huijbers E.J.M., Van Der Werf I.M., Faber L.D., Sialino L.D., Van Der Laan P., Holland H.A., Cimpean A.M., Thijssen V.L.J.L., Van Beijnum J.R., Griffioen A.W. (2019). Targeting Tumor Vascular CD99 Inhibits Tumor Growth. Front. Immunol..

[3] van Beijnum J.R., Huijbers E.J.M., van Loon K., Blanas A., Akbari P., Roos A., Wong T.J., Denisov S.S., Hackeng T.M., Jimenez C.R. (2022). Extracellular vimentin mimics VEGF and is a target for anti-angiogenic immunotherapy. Nat. Commun..

[4] van Loon K., Huijbers E.J.M., de Haan J.D., Griffioen A.W. (2022). Cancer Vaccination against Extracellular Vimentin Efficiently Adjuvanted with Montanide ISA 720/CpG. Cancers (Basel).

[5] Blanas A., Karsjens H., de Ligt A., Huijbers E.J.M., van Loon K., Denisov S.S., Durukan C., Engbersen D.J.M., Groen J., Hennig S. (2022). Vaccination with a bacterial peptide conjugated to SARS-CoV-2 receptor-binding domain accelerates immunity and protects against COVID-19. iScience.

[6] Petsch D., Anspach F.B. (2000). Endotoxin removal from protein solutions. J. Biotechnol..

[7] Huijbers E.J.M., Ringvall M., Femel J., Kalamajski S., Lukinius A., Åbrink M., Hellman L., Olsson A.K. (2010). Vaccination against the extra domain-B of fibronectin as a novel tumor therapy. FASEB J.

[8] Myburgh L., Karsjens H., Blanas A., de Ligt A., van Loon K., Huijbers E.J.M., van Beijnum J.R., Engbersen D.J.M., Rekiki A., Mignon C. (2025). Targeting the early life stages of SARS-CoV-2 using a multi-peptide conjugate vaccine. Vaccine.

[9] Meneveau M.O., Kumar P., Lynch K.T., Patel S.P., Slingluff C.L. (2022). The vaccine-site microenvironment: impacts of antigen, adjuvant, and same-site vaccination on antigen presentation and immune signaling. J. Immunother. Cancer.

[10] Engbersen D.J.M., van Beijnum J.R., Roos A., van Beelen M., de Haan J.D., Grinwis G.C.M., Schalken J.A., Witjes J.A., Griffioen A.W., Huijbers E.J.M. (2023). Vaccination against Extracellular Vimentin for Treatment of Urothelial Cancer of the Bladder in Client-Owned Dogs. Cancers (Basel).

