# Supplementary material for: Protocol for recombinant expression in E. coli and purification of iBoost vaccine proteins using immobilized metal affinity chromatography
Source: STAR Protoc. 2026 May 19;7(2):104572. doi: 10.1016/j.xpro.2026.104572 (PMC13213800; doi:10.1016/j.xpro.2026.104572)
Supplement: Data S1. Vector map TRX-His protein [file mmc1.pdf]

**Supplementary information – vector map TRX-His protein**

Below the insert DNA sequence of TRX-His is given into the multiple cloning site of the pET21a (+) plasmid (Novagen).

**pET-21a-TRX-His**

pET-21a – ***Nde*I**, **TRX (342 nt)**, ***Bam*HI**, **His-Tag**, **Stop**, ***Xho*I** – pET-21a

```
CATATGAGCGATAAAATTATTCACCTGACTGACGACAGTTTTGACACGGATGTACTCAAAGC
GGACGGGGCGATCCTCGTCGATTTCTGGGCAGAGTGGTGCGGTCCGTGCAAAATGATCGCCC
CGATTCTGGATGAAATCGCTGACGAATATCAGGGCAAACTGACCGTTGCAAAACTGAACATC
GATCAAAACCCTGGCACTGCGCCGAAATATGGCATCCGTGGTATCCCGACTCTGCTGCTGTT
CAAAAACGGTGAAGTGGCGGCAACCAAAGTGGGTGCACTGTCTAAAGGTCAGTTGAAAGAGT
TCCTCGACGCTAACCTGGCCGGTTCTGGTTCTGGTAGCGGATCCCACCATCACCATCACCAT
TAGCTCGAG
```

**Number of amino acids:** 123

**Molecular weight:** 13205.99 Da (13 kDa)

**Theoretical pI:** 5.71

**TRX PROTEIN SEQEUNCE**
MSDKIIHLTDDSFDTDVLKADGAILVDFWAEWCGPCKMIAPILDEIADEYQGKLTV
AKLNIDQNPGTAPKYGIRGIPTLLLFKNGEVAATKVGALSKGQLKEFLDANLAGSGS
GSGSHHHHHH

PDB: P0AA25 THIO_ECOLI, trxA

Thioredoxin 1 [Escherichia coli CFT073]
Sequence ID: AAN83133.1Length: 144Number of Matches: 1

| Score | Expect | Method | Identities | Positives | Gaps |
|---|---|---|---|---|---|
| 224 bits(571) | 5e-75 | Compositional matrix adjust. | 109/109(100%) | 109/109(100%) | 0/109(0%) |

```
Query  1    MSDKIIHLTDDSFDTDVLKADGAILVDFWAEWCGPCKMIAPILDEIADEYQGKLTVAKLN  60
            MSDKIIHLTDDSFDTDVLKADGAILVDFWAEWCGPCKMIAPILDEIADEYQGKLTVAKLN
Sbjct  36   MSDKIIHLTDDSFDTDVLKADGAILVDFWAEWCGPCKMIAPILDEIADEYQGKLTVAKLN  95

Query  61   IDQNPGTAPKYGIRGIPTLLLFKNGEVAATKVGALSKGQLKEFLDANLA  109
            IDQNPGTAPKYGIRGIPTLLLFKNGEVAATKVGALSKGQLKEFLDANLA
Sbjct  96   IDQNPGTAPKYGIRGIPTLLLFKNGEVAATKVGALSKGQLKEFLDANLA  144
```
